# Seed Morphotype and Population Origin Shape Germination Responses of Three *Salicornia perennans* Willd. subsp. *perennans* Populations Under Different NaCl Concentrations

**DOI:** 10.3390/plants15152285

**Published:** 2026-07-26

**Authors:** Irene Ventura, Tiziana Lombardi

**Affiliations:** 1 Department of Agriculture, Food and Environment, University of Pisa, Via del Borghetto 80, 56124 Pisa, Italy; tiziana.lombardi@unipi.it; 2 Interdepartmental Research Centre for the Study of Climate Change Impacts, University of Pisa, Via del Borghetto 80, 56124 Pisa, Italy

**Keywords:** *Salicornia perennans* Willd., seed heteromorphism, halophytes, salt stress, germination ecology, dose–response modeling, saltmarsh restoration

## Abstract

Successful germination is a critical stage for the establishment and persistence of annual halophytes in Mediterranean saltmarshes, where highly variable salinity creates narrow recruitment windows. Seed heteromorphism is a common feature of annual halophytes and has been proposed to contribute to recruitment under variable environmental conditions. This study investigated how seed morphotype, NaCl concentration and population origin interact to shape germination responses in *Salicornia perennans* Willd. subsp. *perennans*. Seeds from three Tuscan coastal populations (Italy) were separated into two naturally occurring morphotypes (large and small) and exposed to four NaCl concentrations (0, 5, 10 and 20 g L^−1^) in a fully factorial experiment. Seed morphotype emerged as the main determinant of germination performance: large seeds consistently achieved a higher final germination percentage and germination index than small seeds across populations and salinity treatments. Increasing salinity reduced final germination percentage and germination index but had no significant effect on mean germination time or the time required to reach 50% of final germination (T50) among seeds that germinated, which indicates that NaCl acted as a quantitative filter rather than a kinetic brake. Dose–response modeling yielded comparable EC50 estimates among populations for large seeds, where EC50 represents the salinity at which the fitted germination response reaches the midpoint between the lower and upper asymptotes, whereas the maximum germination capacity differed substantially. Unexpectedly, seeds from San Rossore, the site most influenced by freshwater inputs, maintained the highest germination under severe salt stress. Overall, our results identify seed morphotype as the primary determinant of germination success in *S. perennans* and find that it overrides differences among populations in their response to salinity.

## 1. Introduction

Saltmarshes are among the most ecologically productive and functionally complex coastal ecosystems on Earth, but they are also among the most threatened, having lost more than 50% of their global extent over the past century through land reclamation, coastal development and hydrological alteration [1,2]. These ecosystems provide numerous ecological services, including shoreline stabilization, carbon sequestration, nutrient retention and habitat provision for a wide range of organisms. Along the Tyrrhenian coast of central Italy, saltmarsh and brackish wetland remnants persist as fragmented patches within a heavily modified landscape, increasingly threatened by sea-level rise and freshwater extraction from coastal aquifers [3].

The vegetation of saltmarshes is dominated by halophytes, species capable of completing their life cycle under saline conditions through a range of physiological and biochemical adaptations [4,5]. Species richness is generally low, with a limited number of taxa accounting for most of the biomass and primary productivity of these systems [4,6]. Within saltmarshes, plant communities are arranged along gradients of inundation frequency and salinity in recognizable zonation patterns [7]. Along the Tyrrhenian coast, annual species of *Salicornia* L. s.s. occupy lower marsh zones between periodically flooded mudflats and perennial halophytic vegetation dominated by *Salicornia fruticosa* (L.) A. J. Scott and *Juncus maritimus* Lam. This position exposes populations to pronounced temporal fluctuations in salinity, which makes successful germination a critical determinant of population persistence.

Germination is generally considered the most salt-sensitive phase of the halophyte life cycle. The physiological mechanisms underpinning salt tolerance in adult plants are not yet fully operational in germinating seeds, which must cope with osmotic and ionic stress using comparatively limited resources [4,8]. Consequently, even strongly halophytic species usually germinate best under conditions of reduced salinity, with germination percentage progressively declining as salinity increases [9,10,11]. In Mediterranean saltmarshes, winter and early spring rainfall create a narrow but critical recruitment window for annual species [11,12], and the balance between germination, dormancy and mortality during this period can strongly influence population dynamics.

One mechanism through which annual halophytes cope with the unpredictability of this recruitment window is seed heteromorphism, the production by a single individual of morphologically and physiologically distinct seed types. By distributing germination risk among morphotypes differing in dormancy, germination requirements and stress tolerance, heteromorphic species adopt a strategy consistent with the bet-hedging framework [8,13,14]. Within the Salicornioideae, seed heteromorphism is well documented [15,16]. In *Salicornia* spp., larger central seeds generally exhibit higher germination potential and greater tolerance to salinity, whereas smaller lateral seeds tend to be more dormant and more sensitive to environmental stress [16,17,18,19]. For example, in *Salicornia patula* Duval-Jouve, central seeds germinate readily across a broad salinity range, whereas lateral seeds are strongly inhibited by increasing NaCl concentrations [12]. Ecologically, larger seeds are often associated with rapid recruitment under favorable conditions, while smaller seeds may contribute disproportionately to soil seed-bank persistence [8,14,20]. However, the extent to which these differences remain consistent across populations experiencing contrasting environmental conditions remains poorly understood.

Population-level variation adds a further layer of complexity. Differences in germination responses among populations may arise through local adaptation, maternal environmental effects or neutral genetic processes [21,22]. Environmental conditions experienced during seed development can influence seed size, reserve accumulation, dormancy and seed coat properties independently of genetic differentiation [8,23,24,25]. Consequently, populations originating from more stressful habitats do not necessarily produce seeds with superior germination performance under stress. Recent studies on *Salicornia* have revealed substantial variation in germination behavior among populations and genotypes even under common experimental conditions [26,27], suggesting that provenance effects may be as important as salinity itself in shaping recruitment success.

This study addresses three explicit research questions. First, how does the germination performance of *S. perennans* Willd. subsp. *perennans* respond to increasing NaCl concentration across a biologically relevant salinity range? Second, does naturally occurring seed heteromorphism modulate germination responses to salinity, and does this effect vary among populations? Third, do populations from habitats differing in their salinity regime show divergent germination strategies, and if so, are these differences consistent with a simple expectation based on source-habitat salinity, or do they point instead to a role for seed provisioning and maternal environmental effects? By combining a factorial germination experiment with dose–response modeling, we aimed to disentangle the relative contributions of salinity, seed morphotype and population origin to germination performance and to evaluate whether germination responses can be predicted from source-habitat salinity alone.

## 2. Results

### 2.1. Seed Morphometric Traits

**Figure 1:**
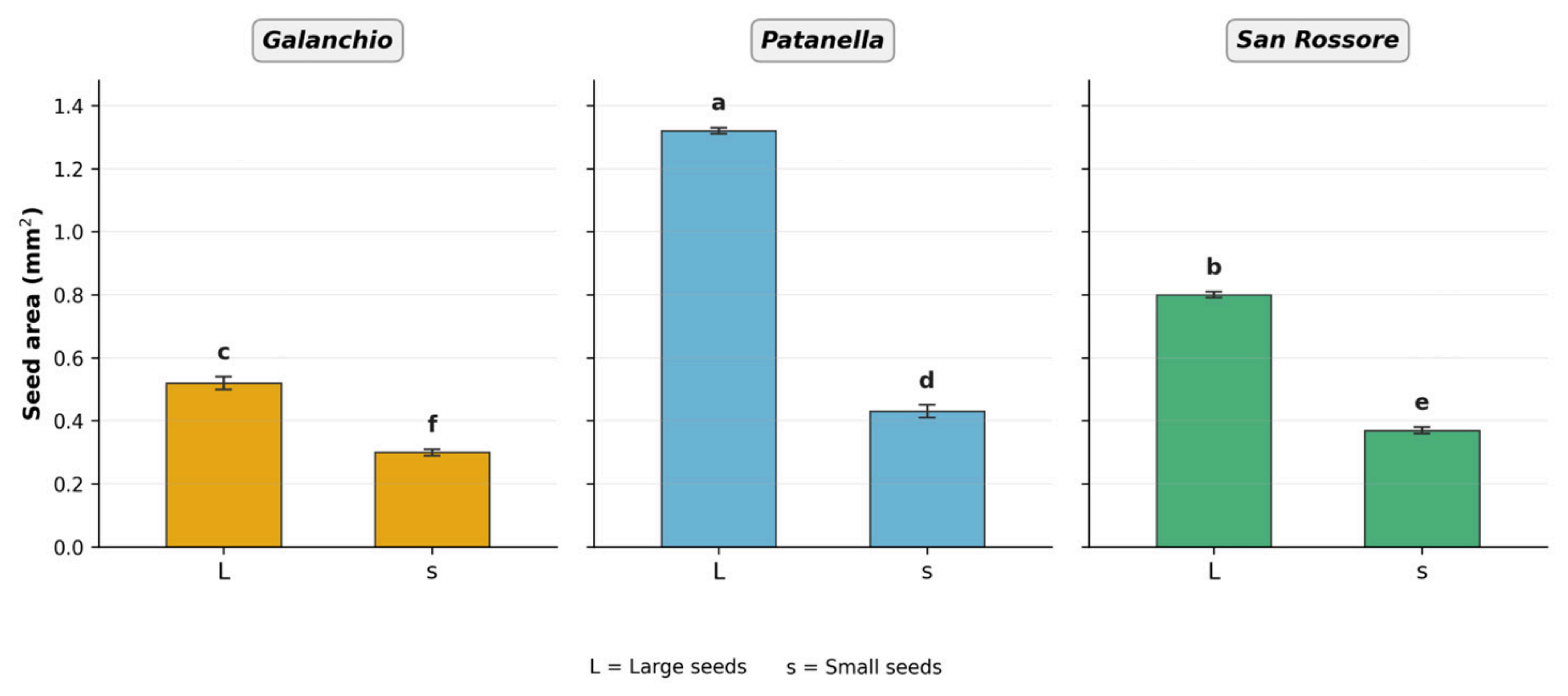
Mean seed area (mm^2^ ± SE) of large (L) and small (s) seeds from the Galanchio, Patanella and San Rossore populations of *Salicornia perennans* Willd. subsp. *perennans* (*n* = 4). Different letters indicate significant differences among population × morphotype combinations according to Tukey’s post hoc test (*p* < 0.05).

Seed area was significantly affected by both seed morphotype (F= 6500.75, df = 1, 18, *p* < 0.001) and population (F = 1385.83, df = 2, 18, *p* < 0.001), with a significant seed × population interaction (F = 255.57, df = 2, 18 *p* < 0.001) (Figure 1). Overall, large seeds exhibited a substantially greater estimated area than small seeds across all populations. Among the large-seed morphotype, seeds from Patanella had the largest estimated area (1.32 ± 0.01 mm^2^), followed by San Rossore (0.80 ± 0.01 mm^2^) and Galanchio (0.52 ± 0.02 mm^2^). A similar pattern was observed for the small-seed morphotype, with Patanella (0.43 ± 0.02 mm^2^) and San Rossore (0.37 ± 0.01 mm^2^) producing significantly larger seeds than Galanchio (0.30 ± 0.01 mm^2^). The significant interaction between seed morphotype and population indicates that the magnitude of seed dimorphism differed among populations. In particular, the difference between large and small seeds was most pronounced in Patanella, where large seeds were approximately three times larger than small seeds, whereas the contrast was less marked in Galanchio and San Rossore. Consequently, Patanella exhibited the strongest expression of seed heteromorphism, while Galanchio produced the smallest seeds regardless of morphotype.

### 2.2. Final Germination Percentage

**Figure 2:**
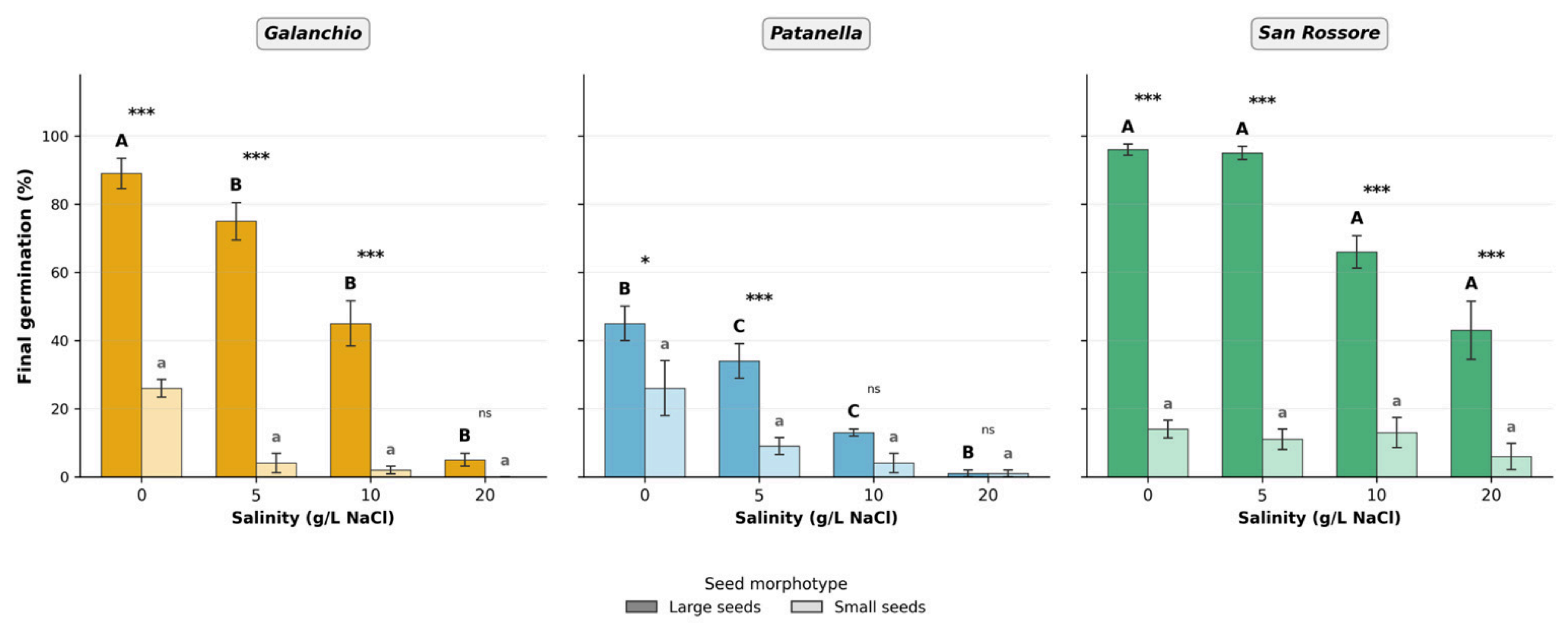
Final germination percentage (FGP, %) of small- and large-seed morphotypes of *Salicornia perennans* Willd. subsp. *perennans* from Galanchio, Patanella and San Rossore under four salinity levels (0, 5, 10 and 20 g L^−1^ NaCl). Values are expressed as means ± SE (*n* = 4). Asterisks indicate significant differences between morphotypes within each provenance × salinity combination (quasibinomial GLM, estimated marginal means, BH-adjusted: *** *p* < 0.001; * *p* < 0.05; ns = not significant). Letters indicate differences among provenances within each salinity level, separately for large (uppercase) and small (lowercase) seeds; bars sharing a letter do not differ significantly. Complete mean-separation results are reported in Table S2, Part F.

Large seeds generally achieved a higher FGP than small seeds across populations and salinity levels (Figure 2; Table S2, Part A). At 0 g L^−1^ NaCl, the mean FGP of large seeds was 89 ± 4%, 45 ± 5% and 96 ± 2% in Galanchio, Patanella and San Rossore, respectively, while small seeds reached 26 ± 3%, 26 ± 8% and 14 ± 3%, respectively. Post hoc comparisons confirmed a significantly higher FGP in large than in small seeds in most provenance × salinity combinations. The only exceptions were Patanella at 10 g L^−1^ NaCl, where the contrast was marginally non-significant (*p* = 0.057), and Galanchio and Patanella at 20 g L^−1^ NaCl, where germination was negligible in both morphotypes (Table S2, Part B).

FGP declined with increasing salinity in all populations and in both morphotypes. Among small seeds, values were low throughout, even at 0 g L^−1^ NaCl; FGP did not exceed 26% in any population and declined to 13% or less at 10 g L^−1^ and 6% or less at 20 g L^−1^. Among large seeds, the salinity response was more pronounced and differed markedly among provenances. Galanchio and Patanella large seeds declined from 89% and 45% at 0 g L^−1^ to 45% and 13% at 10 g L^−1^, respectively, reaching very low values at 20 g L^−1^. San Rossore large seeds showed a more gradual decline, from 96% at 0 g L^−1^ to 95% at 5 g L^−1^, 66% at 10 g L^−1^ and 43% at 20 g L^−1^. Post hoc comparisons confirmed that, among large seeds, San Rossore had a significantly higher FGP than Patanella at 5, 10 and 20 g L^−1^ NaCl (all *p* < 0.001) and a higher FGP than Galanchio at the same salinity levels (*p* ≤ 0.009). Galanchio large seeds outperformed Patanella large seeds at 0, 5 and 10 g L^−1^ NaCl (all *p* < 0.001; Table S2, Part C). Additional post hoc comparisons among all provenance × salinity combinations, performed separately for each seed morphotype, are reported in Table S2, Part F. Among large seeds, all pairwise comparisons among salinity levels were significant in Galanchio, whereas 0 and 5 g L^−1^ NaCl did not differ in Patanella or San Rossore; all remaining within-provenance comparisons were significant. Among small seeds, significant differences were detected between 0 and 5 and between 0 and 10 g L^−1^ NaCl in Galanchio and between 0 and all higher salinity levels in Patanella. No significant differences among salinity levels were detected for small San Rossore seeds.

The statistical analysis confirmed these patterns, showing that FGP was significantly affected by provenance, salinity and seed morphotype, with significant provenance-dependent responses to salinity and seed morphotype differences (Table 1). The GLM identified significant effects of provenance (F = 42.84, df = 2, 72, *p* < 0.001), salinity (F = 74.54, df = 3, 72, *p* < 0.001), seed morphotype (F = 51.11, df = 1, 72, *p* < 0.001), the provenance × salinity interaction (F = 2.36, df = 6, 72, *p* = 0.039), and the provenance × seed morphotype interaction (F = 7.20, df = 2, 72, *p* = 0.001). The salinity × seed morphotype interaction was not significant (F = 1.66, df = 3, 72, *p* = 0.182). The three-way interaction was marginally non-significant (F = 1.89, df = 6, 72, *p* = 0.095).

### 2.3. Germination Index

**Figure 3:**
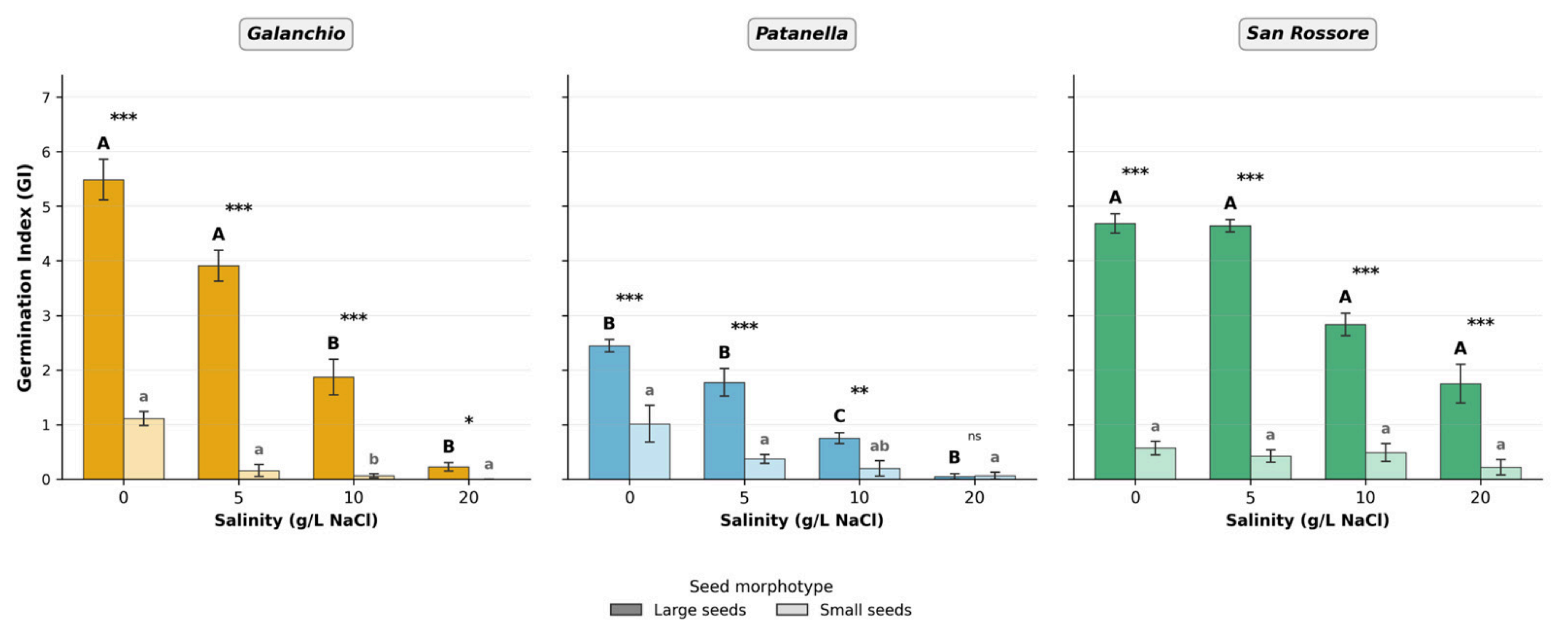
Germination index (GI) of small- and large-seed morphotypes of *Salicornia perennans* Willd. subsp. *perennans* from Galanchio, Patanella and San Rossore under four salinity levels (0, 5, 10 and 20 g L^−1^ NaCl). Values are expressed as means ± SE (*n* = 4). Asterisks indicate significant differences between morphotypes within each provenance × salinity combination (LM on square-root-transformed data, estimated marginal means, BH-adjusted: *** *p* < 0.001; ** *p* < 0.01; * *p* < 0.05; ns = not significant). Letters indicate differences among provenances within each salinity level, separately for large (uppercase) and small (lowercase) seeds; bars sharing a letter do not differ significantly. Complete mean-separation results are reported in Table S2, Part F.

Large seeds generally achieved higher GI values than small seeds across treatment combinations, with the exception of Patanella at 20 g L^−1^ NaCl, where the GI was near zero in both morphotypes (Figure 3; Table S2, Part D). Among large seeds, the mean GI at 0 g L^−1^ NaCl was 5.49 ± 0.37, 2.44 ± 0.11 and 4.68 ± 0.18 in Galanchio, Patanella and San Rossore, respectively, declining to 0.23 ± 0.08, 0.05 ± 0.05 and 1.75 ± 0.35, respectively, at 20 g L^−1^. Among small seeds, GI values were uniformly low across all populations and salinity levels, ranging from 0 to 1.18. Post hoc comparisons confirmed that, among large seeds, San Rossore had a significantly higher GI than Patanella at all salinity levels and a higher GI than Galanchio at 20 g L^−1^ NaCl (Table S2, Part E). Additional post hoc comparisons among all provenance × salinity combinations, performed separately for each seed morphotype, are reported in Table S2, Part F. Among large seeds, all pairwise comparisons among salinity levels were significant in Galanchio, whereas 0 and 5 g L^−1^ NaCl did not differ in Patanella or San Rossore; all remaining within-provenance comparisons were significant. Among small seeds, the GI differed significantly between 0 and all higher salinity levels in Galanchio; between 0 and 10, 0 and 20, and 5 and 20 g L^−1^ NaCl in Patanella; and only between 0 and 20 g L^−1^ NaCl in San Rossore. The pattern observed for GI was broadly consistent with that of FGP, although the seed morphotype effect was particularly pronounced (Table 1). Significant effects were found for provenance (F = 32.68, df = 2, 72, *p* < 0.001), salinity (F = 95.51, df = 3, 72, *p* < 0.001), seed morphotype (F = 401.61, df = 1, 72, *p* < 0.001), provenance × salinity (F = 4.82, df = 6, 72, *p* < 0.001), provenance × seed morphotype (F = 26.32, df = 2, 72, *p* < 0.001), and the salinity × seed morphotype interaction (F = 14.73, df = 3, 72, *p* < 0.001), while the three-way interaction was not significant (F = 1.23, df = 6, 72, *p* = 0.301).

### 2.4. Cumulative Germination Curves

**Figure 4:**
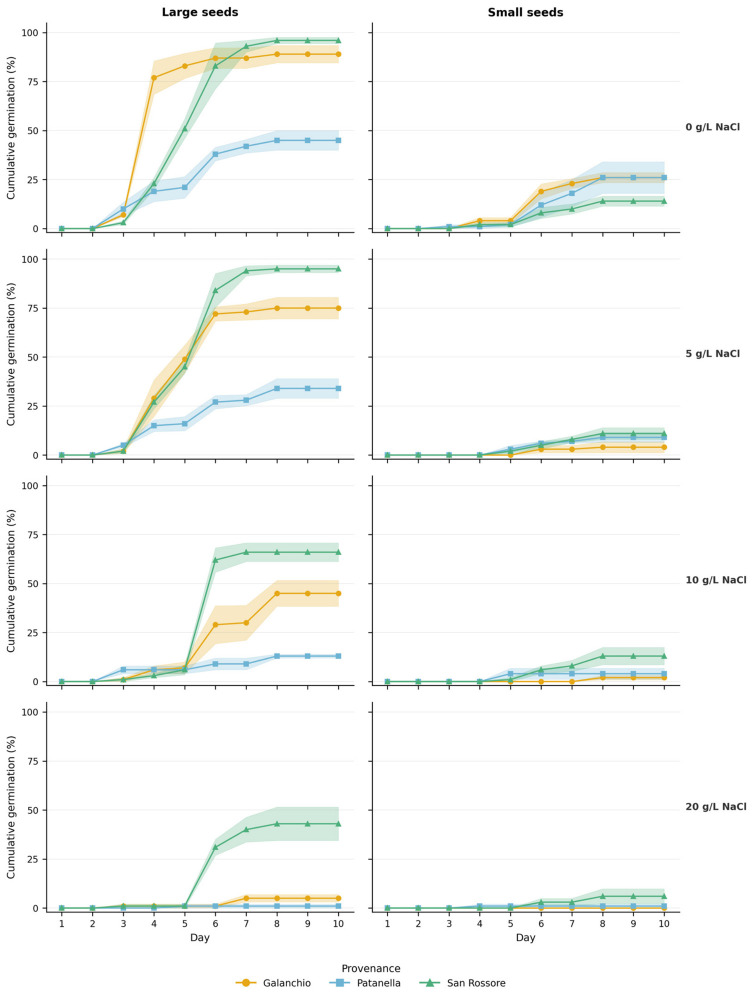
Cumulative germination curves (%) of small- and large-seed morphotypes from three Tuscan coastal populations (Galanchio, Patanella and San Rossore) of *Salicornia perennans* Willd. subsp. *perennans* exposed to four salinity levels (0, 5, 10 and 20 g L^−1^ NaCl). Values represent means ± SE (*n* = 4); shaded ribbons indicate ±1 SE around each curve.

The temporal dynamics of germination are illustrated by the daily cumulative curves (Figure 4). For large seeds at 0 and 5 g L^−1^ NaCl, all three populations reached near-plateau values by days 5–6. At 10 and 20 g L^−1^ NaCl, San Rossore large seeds continued accumulating germination through days 6–7, while Galanchio and Patanella large seeds showed minimal germination beyond day 5. For small seeds, cumulative curves were low and flat across all populations and salinity levels, with most germination events occurring between days 5 and 8.

### 2.5. Mean Germination Time and T50

**Figure 5:**
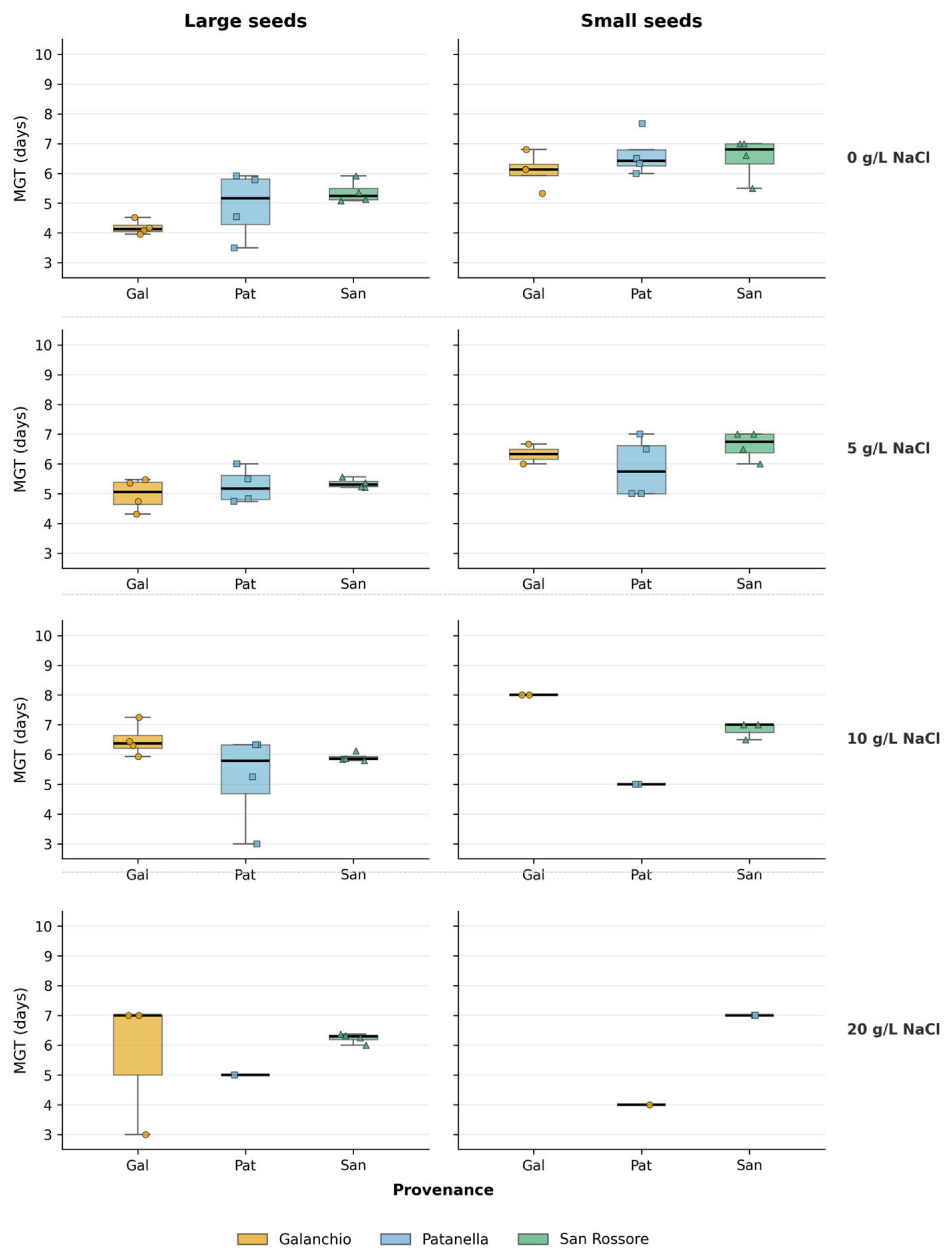
Mean germination time (MGT, days) of large- and small-seed morphotypes of *Salicornia perennans* Willd. subsp. *perennans* from Galanchio, Patanella and San Rossore, under four salinity levels (0, 5, 10 and 20 g L^−1^ NaCl). Boxes show median, interquartile range and 1.5 × IQR; jittered symbols represent individual replicates. Neither salinity nor provenance significantly affected MGT within individual treatment combinations; however, provenance had a marginally significant effect on MGT when pooled across salinity treatments and seed morphotypes (Kruskal–Wallis test: H = 6.01, df = 2, *p* = 0.050), with a significant pairwise difference between San Rossore and Patanella (Dunn’s post hoc test, BH-adjusted, *p* = 0.043). Seed morphotype had a significant effect on MGT (Wilcoxon rank-sum test: W = 1092.5, *p* < 0.001). Pairwise post hoc comparisons among provenances within each specific salinity × morphotype combination were not performed. Because MGT and T50 are defined only for replicates in which germination occurred, cells with predominantly zero germination—itself a meaningful biological outcome already captured in FGP (Figure 2)—necessarily yielded very few or no timeable events, which precluded reliable cell-specific inference for these two variables.

Among the replicates in which germination occurred, large seeds germinated faster, with the mean MGT ranging from 3.96 to 6.48 days depending on provenance and salinity treatment compared to 5.00–8.00 days for small seeds (Figure 5, Table S2, Part A).

Neither MGT nor T50 was significantly affected by salinity (MGT: H = 3.93, df = 3, *p* = 0.269; T50: H = 5.15, df = 3, *p* = 0.161), and T50 was not significantly affected by provenance (H = 3.81, df = 2, *p* = 0.149). Provenance had a marginally significant effect on MGT (H = 6.01, df = 2, *p* = 0.050), with Dunn’s post hoc test identifying a significant difference between San Rossore and Patanella (*p* = 0.043). Seed morphotype had a highly significant effect on both variables (MGT: W = 1092.5, *p* < 0.001; T50: W = 1024, *p* < 0.001).

### 2.6. Dose–Response Modeling and EC50 Estimation

**Figure 6:**
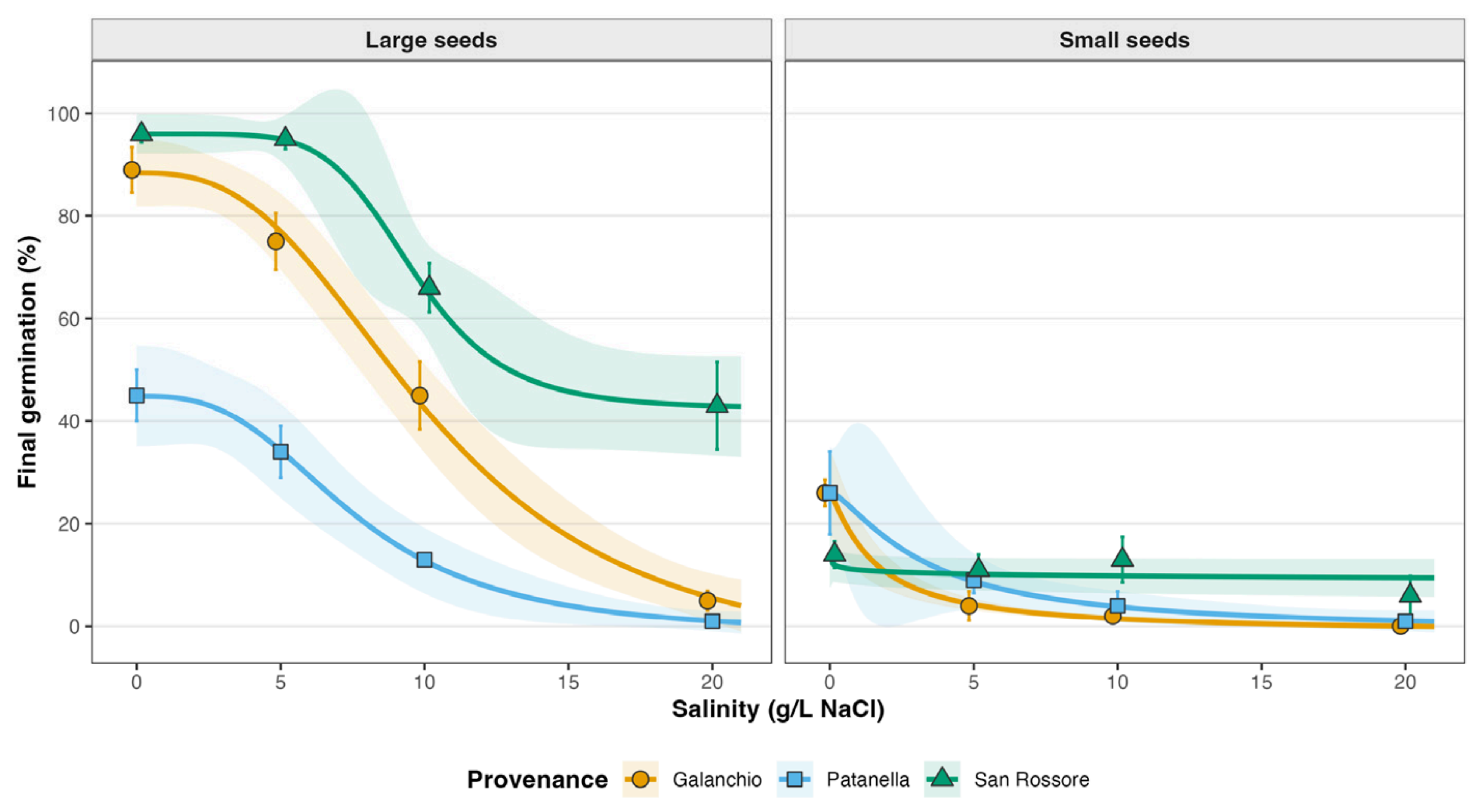
Four-parameter log-logistic (LL.4) dose–response models describing the relationship between salinity and final germination percentage (FGP) for large- and small-seed morphotypes from the Galanchio, Patanella and San Rossore populations of *Salicornia perennans* Willd. subsp. perennans. Symbols represent observed mean values ± SE, solid lines indicate fitted LL.4 curves, and shaded areas represent 95% confidence intervals.

For the large-seed morphotype, the four-parameter log-logistic (LL.4) model converged successfully for all populations and provided reliable parameter estimates (Table 2; Figure 6). The estimated EC50 values were remarkably similar among provenances, ranging from 7.58 to 9.71 g L^−1^ NaCl. Pairwise comparisons revealed no significant differences in EC50 among populations (all *p* > 0.10; Table 2B), indicating comparable salinity sensitivity during germination.

By contrast, substantial differences were observed in the upper asymptote of the dose–response curves (parameter d), which represents the maximum germination capacity. Patanella exhibited a markedly lower upper asymptote (d = 0.45) than Galanchio (d = 0.87) and San Rossore (d = 0.96), which reflects the lower final germination percentages observed across the salinity gradient. These results indicate that population-level differences in germination performance were primarily associated with variation in maximum germination capacity rather than with differences in salinity sensitivity per se.

These results were robust to model specification: comparisons between the four- and three-parameter log-logistic models, and between the log-logistic and Weibull model families, yielded consistent rankings of populations by both EC50 and maximum germination capacity (Table S4).

For the small-seed morphotype, germination remained low across most salinity treatments, which resulted in unstable or biologically poorly informative dose–response parameters. Consequently, the EC50 estimates for small seeds were considered descriptive only and were not used for statistical comparisons among populations (Table 2; Figure 6 and Figure S1). Formal pairwise EC50 comparisons were, therefore, restricted to the large-seed morphotype.

## 3. Discussion

The results of this study provide new insights into how germination in *Salicornia perennans* Willd. subsp. *perennans* is shaped by the interplay of seed morphotype, salinity and population origin. The three factors do not act independently; rather, their interactions define the germination landscape of this species in ways that are ecologically informative and, particularly in the case of San Rossore, not fully predictable from source-habitat salinity alone. Germination assays were conducted under environmental conditions (temperature, photoperiod, and moisture availability) previously identified as optimal for germination in *Salicornia* species [11], thereby minimizing the influence of non-salinity-related constraints on germination. However, seed viability was not assessed using tetrazolium staining or cut tests, and no dormancy-breaking treatments were applied. Consequently, it was not possible to determine whether ungerminated seeds, particularly the small morphotype in the control treatment, were viable but dormant, exhibited delayed germination potential, or were non-viable. This represents a limitation of the present study and precludes direct inference regarding seed dormancy, delayed-germination behavior, or the potential contribution of ungerminated seeds to soil seed-bank persistence. Accordingly, any ecological interpretation of the contrasting germination behavior of the two seed morphotypes should be regarded as a plausible hypothesis rather than direct evidence. Because the viability of the seeds remaining ungerminated at the end of the experiment was not assessed, low germination cannot be attributed specifically to dormancy, aging, loss of viability or mortality occurring during the germination test. The present results, therefore, describe realized germination under the tested conditions and within the experimental time frame, rather than the total viable fraction of each seed lot. From an ecological perspective, ungerminated seeds did not contribute to immediate recruitment during the experimental window; however, their subsequent fate remains unknown, as dormant seeds could potentially contribute to later recruitment, whereas non-viable seeds cannot.

In *Salicornia patula* Duval-Jouve, small lateral seeds have been reported to require cold stratification in addition to light for germination, whereas large central seeds do not [12]. This finding provides one possible interpretation of the low germination observed in the small morphotype, but it does not allow the physiological status of the ungerminated seeds in the present study to be determined. A related limitation concerns the dry-storage period preceding the experiment. Although all seed lots were stored under identical conditions in darkness at ambient temperature for approximately two months, standardized storage cannot be assumed to have produced an identical physiological effect across provenances and seed morphotypes. If the seed lots differed in their initial dormancy status, dry after-ripening may have reduced dormancy to different extents and consequently attenuated or obscured pre-existing differences in germination readiness. Therefore, the present comparisons describe the germination responses expressed after a common storage period and should not be interpreted as direct estimates of dormancy status at the time of seed collection. Nevertheless, because all germination assays were conducted under environmental conditions known to be optimal for *Salicornia europaea* aggr. germination, differences among treatments are unlikely to reflect suboptimal experimental conditions. Rather, they provide a robust, standardized comparison of the germination responses expressed by the two seed morphotypes under increasing salinity after a common storage period.

The most striking result of this study was the consistent advantage of large seeds over small seeds across populations and salinity treatments. Large seeds achieved a significantly higher final germination percentage (FGP) and germination index (GI) in almost all provenance × salinity combinations, which demonstrates that seed morphotype is the primary determinant of germination performance rather than a secondary source of variation. The particularly strong effect observed for GI indicates that large seeds not only germinated in greater proportions but also produced a more rapid and synchronized germination response. This pattern is consistent with previous studies on seed heteromorphism in *Salicornioideae*. In *Salicornia europaea* L., larger central seeds exhibit higher germination percentages and greater tolerance to salinity than smaller lateral seeds [17,19]. Similarly, Grouzis et al. (1976) [12] reported that large seeds of *S. patula* germinated readily across a broad salinity range, whereas small seeds were strongly inhibited by increasing NaCl concentrations. More recently, Ameixa et al. (2016) [28] observed contrasting germination responses between central and lateral seeds of *Salicornia ramosissima* J. Woods, confirming that seed dimorphism can strongly influence germination success under saline conditions. Our results extend these observations to *S. perennans* and, to our knowledge, provide the first evidence that seed morphotype remains a dominant driver of germination performance across multiple populations from Tuscany when analyzed simultaneously with provenance and salinity. The mechanistic basis of this advantage may be related to differences in seed provisioning. Larger heteromorphic seeds frequently contain greater reserves of storage compounds, including lipids and proteins, which can support embryo metabolism during imbibition and early radicle emergence under osmotic stress [8]. Accordingly, the higher FGP and GI observed in large seeds of *S. perennans* are consistent with a better-provisioned recruitment strategy. In addition, differences in seed coat permeability may influence the rate of ion uptake during imbibition, thereby modulating the exposure of the embryo to osmotic and ionic stress [20]. Although these traits were not directly measured in the present study, they provide plausible physiological mechanisms underlying the observed differences. Further support for persistent morphotype-dependent physiological divergence comes from *Arthrocnemum macrostachyum* (Moric.) Piirainen & G. Kadereit, where seedlings originating from different seed morphotypes exhibited contrasting antioxidant responses under saline conditions [29]. Interestingly, the strong morphotype effect observed here contrasts with recent findings in *S. brachiata* Roxb., where seed type did not exert a significant main effect on germination, although it interacted with other factors such as seed source and dormancy-breaking treatments [26]. This discrepancy suggests that the ecological significance of seed heteromorphism may vary among *Salicornia* species and populations and highlights the importance of evaluating seed morphotype explicitly rather than treating seed lots as homogeneous units.

From an ecological perspective, the contrasting behavior of the two morphotypes is consistent with a bet-hedging strategy proposed for heteromorphic seeds in annual halophytes [14]. Large seeds, characterized by high and rapid germination, appear adapted to maximize recruitment during the short period of reduced surface salinity that follows winter rainfall in Mediterranean saltmarshes. Small seeds, by contrast, maintained low germination under both saline and non-saline conditions. Rather than simply reflecting inferior seed quality, this behavior may indicate increased dormancy or delayed-germination tendencies, as reported for heteromorphic seeds of several halophytes [8,20]. However, because seed viability and dormancy status were not directly assessed in the present study, it is not possible to distinguish between dormant, delayed-germinating and non-viable seeds. If a substantial proportion of ungerminated small seeds remained viable, their low immediate germination could contribute to prolonged persistence in the soil seed bank and distribute recruitment across years with contrasting environmental conditions, as proposed for other heteromorphic halophytes [14,20]. Consequently, the two seed morphotypes may be viewed as complementary components of a single adaptive strategy rather than as superior and inferior propagules. Nevertheless, this interpretation remains hypothetical and requires direct experimental confirmation through seed viability, dormancy and seed-bank persistence studies.

A second finding that emerged consistently across populations and seed morphotypes was the decoupling between the effects of salinity on germination success and germination timing. Increasing the NaCl concentration progressively reduced FGP and GI, whereas MGT and T50 remained largely unaffected among seeds that successfully germinated. Similar patterns have been reported in *S. europaea*, where salinity significantly reduced germination percentage and vigor-related indices but had little effect on timing-related variables [11]. Our results extend these observations to multiple *S. perennans* populations of Tuscany and suggest that germination success is more sensitive to salinity than germination kinetics.

This pattern has important ecological implications. In Mediterranean coastal wetlands, the germination window is narrow and highly dynamic, being constrained not only by salinity but also by increasing temperatures and the progressive drying of surface soils as spring advances [10,11]. Under such conditions, the absence of a significant salinity effect on MGT and T50 suggests that NaCl did not progressively delay germination among responsive seeds. Instead, salinity appeared to function primarily as an osmotic threshold: seeds either crossed the physiological threshold required for radicle emergence and germinated within a broadly similar time frame or remained ungerminated as osmotic stress increased. In this sense, salinity acted less as a kinetic brake and more as a quantitative filter determining the fraction of seeds able to complete germination. This interpretation is consistent with studies on other *Salicornia* species. Ameixa et al. (2016) [28] reported that increasing salinity reduced final germination in *S. ramosissima* while having comparatively limited effects on germination rate. Similarly, Jacob et al. (2020) [30] showed that seeds of *S. brachiata* retained full viability under high salinity and exhibited complete recovery germination once the osmotic constraint was removed.

Taken together, these findings suggest that the primary ecological effect of salinity during germination may be to regulate the proportion of seeds that germinate under a given set of environmental conditions, rather than simply delaying germination in all seeds. If ungerminated seeds remain viable, such a mechanism could contribute to persistence in highly variable saltmarsh environments by allowing recruitment to occur when more favorable hydrological conditions arise. The third set of findings is particularly noteworthy because it distinguishes differences in maximum germination capacity from detectable differences in salinity sensitivity. Population of origin emerged as a significant source of variation in both FGP and GI, both as a main effect and through its interactions with salinity and seed morphotype. Similar provenance-dependent germination responses have recently been reported for *S. brachiata*, where seeds collected from different coastal regions exhibited significantly different germination capacities under identical experimental conditions [26], and for *S. europaea* aggr., where genotype-dependent variation significantly affected germination performance [27]. In the present study, however, dose–response modeling revealed that the EC50 estimates for large seeds were statistically indistinguishable among the three populations, with values of 9.71, 7.58 and 9.63 g L^−1^ NaCl for Galanchio, Patanella and San Rossore, respectively. Thus, the estimated salinity corresponding to the midpoint between the lower and upper asymptotes of each population’s fitted response curve did not differ significantly among provenances. Population differentiation was instead expressed primarily through variation in the upper asymptote of the dose–response curve, the parameter *d*, which represents the maximum germination capacity. Galanchio, Patanella and San Rossore exhibited estimated *d* values of 0.87, 0.45 and 0.96, respectively, which indicates that populations differed mainly in the proportion of seeds capable of germinating rather than in the salinity level at which inhibition occurred. The superior performance of the San Rossore population should not be interpreted as evidence of greater salinity tolerance *sensu stricto*. Rather, San Rossore maintained a consistently higher germination capacity across the entire salinity gradient despite exhibiting an EC50 comparable to those of the other populations. Consistently, large seeds from San Rossore retained 43% FGP at 20 g L^−1^ NaCl, whereas germination declined to 5% and 1% in Galanchio and Patanella, respectively. These results, therefore, indicate that population-level differences were primarily associated with variation in maximum germination capacity rather than with a detectable shift in salinity sensitivity. However, the positive lower asymptote estimated for large seeds from San Rossore (c = 0.424) should not be interpreted literally as indicating that approximately 42% of seeds would germinate at arbitrarily high salinity. Rather, it likely reflects the fact that the tested salinity range, which extended to 20 g L^−1^ NaCl, did not capture the complete decline of the germination response towards zero. Some halophytic species, including species of Salicornia, have been reported to retain some germination capacity in full-strength seawater with a salinity of approximately 35 PSU [23], which suggests that germination may persist beyond the maximum concentration tested here. Nevertheless, the response of the San Rossore population beyond 20 g L^−1^ remains experimentally unknown. Moreover, fixing the lower asymptote at zero increased the estimated EC50 from approximately 9.6 to 16.64 g L^−1^ (Table S4), which indicates that the absolute EC50 estimate for this population is sensitive to model specification. Additional experiments including higher salinity levels would, therefore, be required to determine the complete shape of the dose–response curve and obtain a more robust estimate of its lower asymptote. This result does not support the simple expectation that populations originating from more saline habitats necessarily exhibit higher germination under saline conditions. The three populations occupy environments that differ substantially in hydrological regime and seasonal salinity dynamics, although a direct quantitative comparison is constrained by the limited availability of homogeneous salinity data collected in the same season, matrix and microhabitat. San Rossore is characterized by strong freshwater influence from the surrounding coastal forest and by seasonally flooded retrodunal depressions, although published conductivity data and recent unpublished measurements indicate that ecologically relevant edaphic salinity may still occur in this system [7]. Galanchio appears to be a highly variable brackish wetland, with published EC values ranging from very low to high values across seasons and recent spring measurements indicating high soil conductivity at the study site [7] (unpublished data). Patanella, located within the confined Orbetello Lagoon and subject to stronger marine influence and marked summer drought, is expected to experience more persistent salinity pressure, although the available published and unpublished data are not directly comparable with those from the other sites because they refer to different periods and measurement contexts [31] (T. Lombardi, unpublished data). Consequently, although the three sites differ markedly in their hydrology and salinity regime, they cannot be arranged along a simple and directly comparable salinity gradient. Under a straightforward local-adaptation hypothesis, populations originating from habitats exposed to stronger or more persistent salinity stress, particularly Galanchio and Patanella, would be expected to exhibit superior germination performance under saline conditions [21,22,32]. Our results do not support this expectation.

The pattern observed here contrasts with that reported by Redondo-Gómez et al. (2007) [33], who found that the germination responses of three perennial *Salicornia* taxa closely reflected their position along a tidal salinity gradient, with the upper marsh taxon *S. fruticosa* (L.) L. maintaining higher germination under hypersaline conditions than taxa occupying lower marsh habitats. It should be noted, however, that Redondo-Gómez et al. (2007) [33] compared distinct perennial taxa rather than populations of the same annual species and that interspecific differences in salinity tolerance may not follow the same rules as intraspecific provenance variation. Comparable complexity has also been reported in other halophytes. For example, Ludwiczak et al. (2024) [25] found that populations of *Tripolium pannonicum* Jacq. originating from contrasting saline habitats differed markedly in their germination behavior and salinity responses, but these differences reflected parental and population-specific effects rather than a straightforward relationship with habitat salinity. Together, these studies, including the present work, suggest that the relationship between habitat salinity and germination performance is not universal and may vary among species, populations and ecological contexts.

Two non-mutually exclusive explanations may account for the patterns observed in *S. perennans*. The first involves maternal environmental effects. Plants growing in the hydrologically buffered environment of San Rossore may have produced seeds with higher physiological quality or more favorable reserve composition, which could have resulted in greater germination capacity under stress [8,24]. Conversely, plants growing in the more stressful lagoonal environment of Patanella may produce seeds whose germination potential is constrained by factors not captured by seed size alone, such as reserve composition, dormancy status, seed coat properties or maternal effects acting during seed development [8,20]. This interpretation is consistent with the broader framework of maternal effects on seed traits in halophytes [25,34] and with the markedly lower upper asymptote observed for Patanella despite its more marine-influenced habitat. Interestingly, the Patanella population produced the largest seeds in both morphotypes yet exhibited the lowest germination capacity, particularly within the large-seed morphotype. This pattern indicates that seed size alone cannot account for the observed differences among populations. Consequently, although larger seeds are generally associated with enhanced germination performance, their ecological advantage appears to depend on additional physiological or maternal factors that remain to be investigated.

A second explanation involves standing genetic variation among populations that is not directly linked to habitat salinity. Founder effects, restricted gene flow, or selection imposed by environmental factors other than salinity, such as inundation regime, soil texture or interspecific competition, may all contribute to population differentiation in germination behavior [6,21]. Because seeds were collected from natural populations during a single reproductive season, the present experimental design does not allow maternal environmental effects to be separated from genetically based population differentiation. Distinguishing between maternal environmental effects and genetically based differentiation will require common-garden experiments across multiple generations, ideally combined with biochemical and structural characterization of the two seed morphotypes.

The practical relevance of these findings extends beyond basic ecophysiology. Restoration projects involving *S. perennans* along the Tyrrhenian coast should not assume that seeds from more marine-influenced or apparently more saline habitats will necessarily perform better under saline germination conditions. Our results indicate that provenance selection should be supported by empirical germination testing rather than inferred from habitat salinity alone. Under the tested conditions, large seeds showed a clear advantage in germination performance. However, the broader literature on seed heteromorphism in halophytes suggests that small seeds may contribute to population persistence through complementary functions not assessed here, including enhanced dispersal, delayed recruitment, and prolonged persistence in the soil seed bank [8,14,20].

## 4. Materials and Methods

### 4.1. Study Species

*Salicornia perennans* Willd. subsp. *perennans* is an annual, succulent halophyte belonging to the family Amaranthaceae (subfam. Salicornioideae).

**Figure 7:**
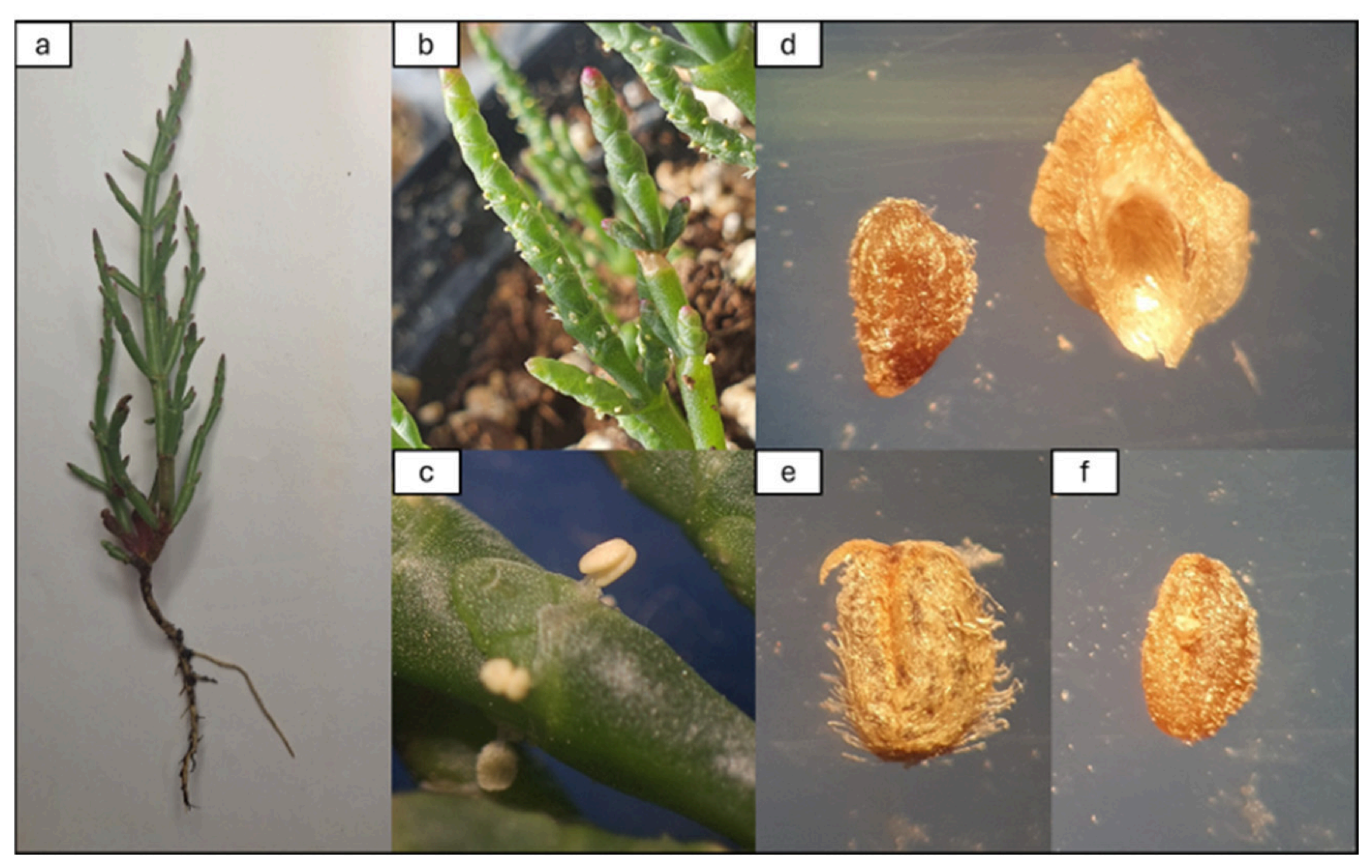
Morphological features of *Salicornia perennans* Willd. subsp. *perennans* (**a**) Representative individual from the Patanella population (Grosseto, Italy); (**b**) fertile shoot bearing the inflorescence; (**c**) detail of the inflorescence showing flowers and stamens; (**d**) dry fruit and enclosed seed; (**e**) large-seed morphotype (morphotype 1); (**f**) small-seed morphotype (morphotype 2). All photographs were taken by the authors (panels (**a**–**c**): June 2025; panels (**d**–**f**): November 2023).

The species is widely distributed across Mediterranean, Black Sea and Central Asian coastal wetlands and represents one of the characteristic annual pioneers of saltmarsh communities along the Italian Tyrrhenian coast [7,35,36,37]. Plants are characterized by green, jointed, succulent stems up to 40 cm in height, with lignification occurring at the stem base; fertile stems turn red at flowering, which occurs in late summer. Flowers are arranged in triads at each node of the fertile segments, with one central and two lateral flowers, each producing a single seed enclosed in a dry indehiscent fruit (Figure 7).

As documented in other species of the *S. europaea* aggregate, *S. perennans* produces two morphologically distinct seed types: a larger, lighter-colored morphotype associated with the central flower, and a smaller, darker morphotype associated with the lateral flowers [17,18].

### 4.2. Study Sites and Seed Collection

**Figure 8:**
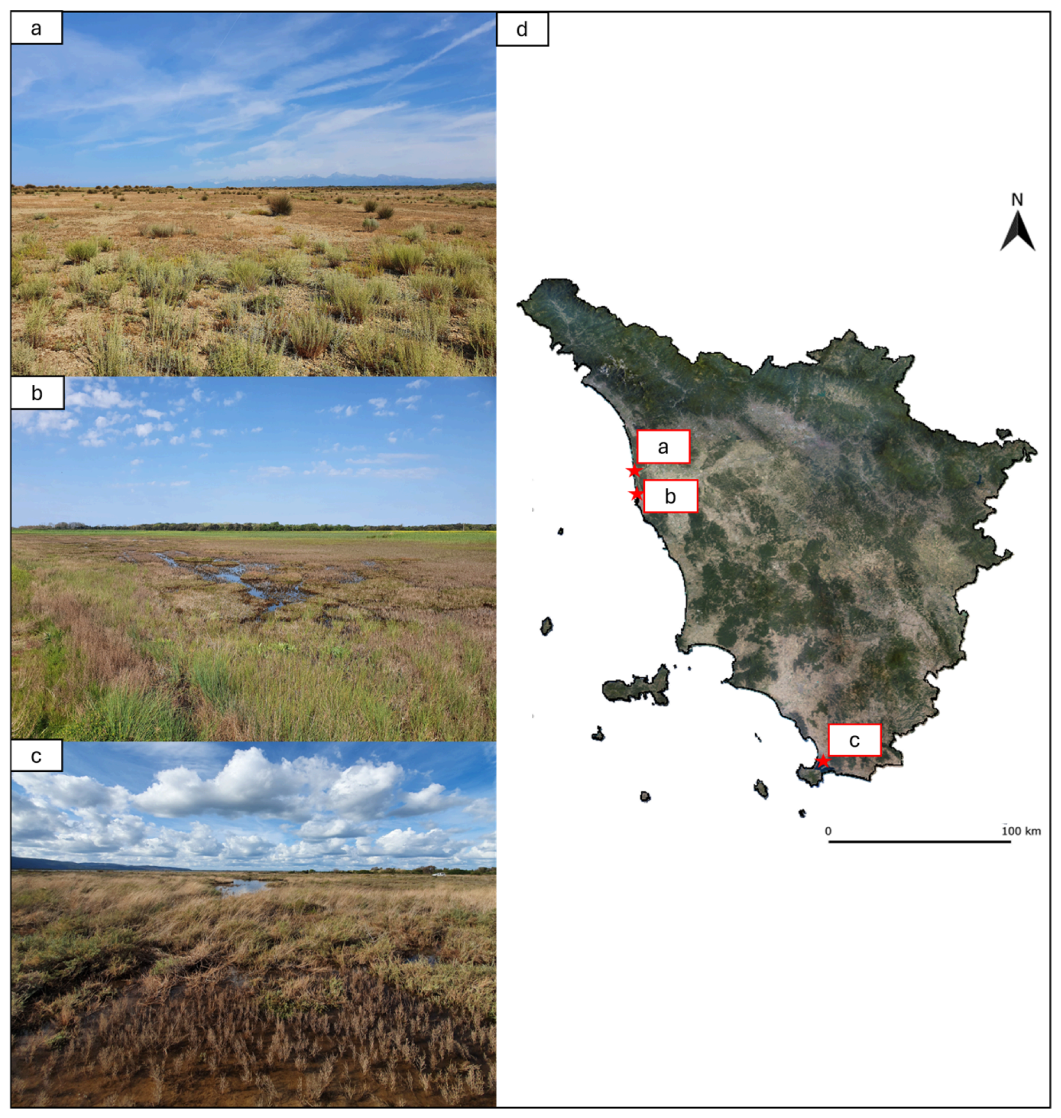
Study sites of *Salicornia perennans* Willd. subsp. *perennans* populations along the Tuscan Tyrrhenian coast, Italy. (**a**) Lame di San Rossore Regional Park, retrodunal saltmarsh; (**b**) Galanchio coastal wetland; (**c**) Patanella saltmarsh within the Orbetello Lagoon system; (**d**) geographic location of the three sampling sites in Tuscany. All photographs in panels (**a**–**c**) were taken by the authors in November 2023. The map (panel (**d**)) was created by the authors using QGIS v3.24.1,QGIS Development Team, (2022) QGIS Geographic Information System. Open Source Geospatial Foundation Project. https://qgis.org/it/, accessed on 23 July 2026) based on the orthophoto available through the Web Map Service (WMS) of the Regione Toscana Geoportal https://www.regione.toscana.it/servizi-wms (accessed on 23 July 2026).

Seeds were collected at maturity in November 2023 from three wild populations of *Salicornia perennans* subsp. *perennans* located along the Tuscan Tyrrhenian coast, Italy (Figure 8). The three populations represent distinct coastal wetland typologies differing in hydrological regime, freshwater influence and marine-lagoonal connectivity.

Climatic data were obtained from the historical archive of the Regional Hydrological Service of Tuscany (Servizio Idrologico Regionale, SIR Toscana; www.sir.toscana.it) using the nearest available meteorological station to each study site: Bocca d’Arno for Lame di San Rossore, Coltano for Galanchio, and Orbetello for Patanella. Daily precipitation and temperature records were aggregated to annual and monthly values over the common period 2013–2024. Monthly climate data for each station are reported in Table S3.

Available soil salinity or conductivity data were used as contextual environmental information only, because they derive from previous studies and may differ among sites in sampling date, season, microhabitat and measurement method.

- Lame di San Rossore (43.686014° N, 10.288099° E)—Site located within the Tenuta di San Rossore, part of the Regional Natural Park of Migliarino, San Rossore e Massaciuccoli (Province of Pisa) (Figure 8a). The site consists of retrodunal interdune depressions locally known as “lame”, characterized by shallow, seasonally flooded brackish water bodies. In this system, salinity is strongly influenced by seasonal precipitation, freshwater inputs from the surrounding coastal forest and the local hydrological dynamics of the retrodune wetland. The nearest climatic reference station was Bocca d’Arno. Over the 2013–2024 period, mean annual precipitation was 910 ± 236 mm, with November as the wettest month on average (133 mm) and June as the driest month (31 mm). Mean annual temperature was 15.9 °C, with a mean annual maximum temperature of 19.8 °C and a mean annual minimum temperature of 12.1 °C. July and August were the warmest months on average, both with a mean temperature of 24.1 °C; August had the highest mean maximum temperature, 28.2 °C. Published soil conductivity data from the Lame area report a wide seasonal range, from 4 to 17 dS/m between autumn and summer, suggesting pronounced temporal variability and possible late-season peaks in edaphic salinity [7]. More recent measurements conducted in April 2025 recorded a soil conductivity of 8.2 dS/m at the study site (unpublished data).
- Galanchio (43°35.467′ N, 10°18.984′ E)—Area located in the southern sector of the Regional Natural Park of Migliarino, San Rossore e Massaciuccoli, at the boundary between the Pisan coastal plain and the industrial-port area of Livorno (Figure 8b). The site is a coastal brackish wetland subject to seasonal fluctuations in water availability and salinity, mainly driven by precipitation, evapotranspiration and the hydrological dynamics of the surrounding coastal plain. The nearest climatic reference station was Coltano. Over the 2013–2024 period, mean annual precipitation was 879 ± 196 mm, very similar to that recorded at Bocca d’Arno. November was the wettest month on average (126 mm), whereas July was the driest month (34 mm), which indicates a pronounced summer rainfall minimum. Mean annual temperature was 15.9 °C, with a mean annual maximum temperature of 21.1 °C and a mean annual minimum temperature of 10.7 °C. August was the warmest month, with a mean temperature of 24.8 °C and a mean maximum temperature of 31.1 °C, which reflects the slightly more inland position of the station within the coastal plain. Published soil conductivity data from the Galanchio area report seasonal EC values ranging from 0.2 to 28 dS/m between autumn and summer, indicating marked temporal variability in edaphic salinity and the occurrence of high conductivity values [7]. More recent measurements conducted in April 2025 recorded a soil conductivity of 22 dS/m at the study site (unpublished data), confirming that high edaphic salinity may also occur during the spring recruitment period.
- Patanella (42°27′22.6″ N, 11°13′44.1″ E)—Site located on the western shore of the Orbetello Lagoon (Province of Grosseto), a shallow coastal lagoon of approximately 27 km^2^ characterized by a confined hydrological regime and restricted water exchange with the sea (Figure 8c). Compared with the two Pisan coastal sites, this setting is more strongly marine-influenced and subject to marked summer drought. The nearest climatic reference station was Orbetello. Over the 2013–2024 period, mean annual precipitation was 508 ± 185 mm, substantially lower than at Bocca d’Arno and Coltano. July and August were the driest and warmest months on average, both with 7 mm of precipitation and a mean temperature of 25.2 °C; August showed the highest mean maximum temperature, 28.0 °C. Mean annual temperature was 17.0 °C, with a mean annual maximum temperature of 19.6 °C and a mean annual minimum temperature of 14.3 °C, which indicates a warmer coastal-lagoonal climate. Overall, the lower rainfall, marked summer drought and confined lagoonal setting support the interpretation of Patanella as the most xeric and marine-influenced of the three study sites. Historical soil data from halophytic stands in the Orbetello Lagoon reported soil salinity values ranging from 0.56 g/kg in March to 3.0 g/kg in May [31]. More recent measurements conducted in October 2025 recorded a soil conductivity of 16 dS/m at the study site (unpublished data), suggesting substantially higher salinity in the post-summer period. These values were used as contextual evidence only, because they derive from different seasons and measurement methods and do not refer specifically to the exact seed-collection microsite.

At each site, ripe infructescences were collected from a minimum of 20 reproductive individuals randomly sampled within each population, and seeds were pooled to obtain a representative bulk sample for each provenance.

It should be noted that seeds from each population were collected as a single bulk sample in a single collection event. This sampling approach is standard in population-level germination ecology but constitutes a single collection replicate per population; population-level conclusions should, therefore, be interpreted as representative of the sampled material rather than generalized to the full genetic and phenotypic diversity of the populations.

Seeds were manually extracted from infructescences, cleaned of plant debris, and separated into the two morphotypes under a stereoscopic microscope based on differences in size and color: large seeds, lighter in color, and small seeds, darker in color. Separated seeds were stored dry in a climatic chamber, with low relative humidity, in the dark and at room temperature (20–23 °C) for approximately two months, until the start of the germination experiment.

### 4.3. Seed Morphometric Measurement

To quantitatively characterize seed heteromorphism in *S. perennans*, morphometric measurements were performed on both seed morphotypes from the three study populations. Seeds were photographed in Petri dishes before the start of the germination experiment using a digital camera under uniform illumination. Four replicates of 25 seeds were measured for each population × morphotype combination, for a total of 600 seeds. Prior to imaging, seeds were manually separated to avoid overlap and positioned on a homogeneous background. Digital images were analyzed using ImageJ software version 1.54g (National Institutes of Health, Bethesda, MD, USA), with scale calibrated using the known Petri dish diameter (90 mm). Images were converted to 8-bit grayscale, and threshold segmentation was applied to isolate seeds from the background. Three parameters were recorded for each seed: maximum seed length (Feret diameter, mm), seed width (minimum Feret diameter, mm) and projected seed area (mm^2^). Seed area was used as the main descriptor of seed size in subsequent analyses. Individual seed measurements were averaged at the Petri dish level, and replicate means were used for statistical comparisons, keeping the Petri dish as the experimental unit.

### 4.4. Germination Experiment

Germination tests were initiated in January 2024. For each combination of population (3), seed morphotype (2) and salinity treatment (4), four replicates of 25 seeds were prepared, for a total of 96 experimental units. Each Petri dish was considered an experimental unit.

Seeds were placed in 9 cm Petri dishes on two layers of Whatman No. 2 filter paper moistened with 9 mL of NaCl solution. Four salinity levels were tested—0, 5, 10 and 20 g L^−1^ NaCl—corresponding to 0, 85.6, 171.1 and 342.2 mM NaCl, respectively, prepared by dissolving analytical-grade NaCl in deionized water. Petri dishes were sealed with Parafilm to minimize evaporation and placed in a growth chamber (University of Pisa) under a 16 h light/8 h dark photoperiod at a photosynthetic photon flux density of 200 µmol m^−2^ s^−1^ (400–700 nm, LED tubes) and a constant temperature of 25 °C.

Seeds were considered germinated upon emergence of the radicle to a minimum length of 2 mm. Germination counts were performed daily. The experiment was terminated when no new germination was observed for three consecutive days, as this criterion was considered indicative of the completion of the germination process under the imposed treatments. No surface sterilization or hormonal pre-treatments were applied to seeds prior to the experiment.

### 4.5. Germination Parameters

Four germination parameters were calculated for each replicate. Final germination percentage (FGP, %) was calculated as:

FGP = (N/Nt) × 100

where N is the total number of germinated seeds at the end of the experiment and Nt is the total number of seeds tested per replicate (25).

The germination index (GI) was calculated using the following formula:

GI = Σ (ni/ti)

where ni is the number of seeds newly germinated on day i and ti is the number of days from the start of the experiment to day i. Higher GI values indicate faster and more concentrated germination.

Mean germination time (MGT, days) was calculated as:

MGT = Σ(ni × ti)/Σni

where ni and ti are as defined above. MGT was calculated only for replicates in which at least one seed germinated; replicates with zero germination were excluded from MGT analyses.

Time to 50% germination (T50, days) was estimated by linear interpolation between the two consecutive daily cumulative counts bracketing 50% of the final germination count:

T50 = Ti + (N/2 − Ni) (Tj − Ti)/(Nj − Ni)

where N is the final number of germinated seeds and Ni and Nj are cumulative germination counts at times Ti and Tj, respectively, with Ni < N/2 < Nj. T50 was calculated for all replicates in which at least one seed germinated. For replicates with a single germinated seed, T50 was estimated as the interpolated time at which 0.5 germinated seeds would have been reached, corresponding to the midpoint between the day preceding germination and the day on which germination was first recorded. Replicates with zero germination were excluded from T50 analyses.

The relationship between NaCl concentration and final germination percentage was further characterized by fitting a four-parameter log-logistic model (LL.4) to the raw replicate data using the drm() function of the drc package [38]. The model describes the dose–response curve as:

f(x) = c + (d − c)/[1 + exp(b · (ln x − ln e))]

where b is the slope at the inflection point, c is the lower asymptote, d is the upper asymptote representing maximum germination capacity, and e is the EC50, corresponding to the salinity at which the fitted response reaches the midpoint between the lower and upper asymptotes. A small constant (0.001 g L^−1^) was added to all salinity values prior to log transformation to accommodate the control treatment at zero salinity. Models were fitted separately for each provenance and seed morphotype combination using binomial weights corresponding to 25 seeds per replicate. Confidence intervals for EC50 were estimated by the delta method. To test for significant differences in EC50 among provenances within each seed morphotype, grouped models were fitted with provenance as a curve identifier, and pairwise EC50 ratios were tested against the null hypothesis of equality using compParm(). Where model convergence failed or variance could not be estimated due to near-zero germination across all salinity levels, EC50 is reported as not estimable and the corresponding population was excluded from pairwise comparisons. To assess model robustness, the LL.4 model was additionally compared against a three-parameter alternative with the lower asymptote fixed at zero (LL.3) and against a Weibull-type model (W1.4) representing a distinct functional form; results are reported in Table S4.

### 4.6. Statistical Analysis

All analyses were performed in R version 4.6.0 (R Core Team, 2026; https://www.r-project.org). Differences in seed area between morphotypes and among populations were assessed using a two-way ANOVA with seed morphotype and population as fixed factors, followed by Tukey’s HSD post hoc test. The effect of provenance, salinity and seed morphotype on FGP was analyzed using a generalized linear model (GLM) with quasibinomial error distribution to account for overdispersion in the proportion data. The response variable was the binomial count of germinated and non-germinated seeds per Petri dish, specified as cbind (germinated, non-germinated), with provenance, salinity, seed morphotype and all their interactions as fixed effects. Significance of fixed effects was assessed using Type III F-tests (car package) [39]. Post hoc pairwise comparisons were performed using estimated marginal means (emmeans package) [40]. Three complementary sets of contrasts were considered—(i) differences between seed morphotypes within each provenance × salinity combination; (ii) differences among provenances within each salinity × seed morphotype combination; and (iii) differences among all 12 provenance × salinity combinations—analyzed separately for each seed morphotype. For the third analysis, all 66 pairwise contrasts were evaluated within each seed morphotype and for each response variable, and compact-letter displays were generated to summarize mean separation (Table S2, Part F). *p*-values were adjusted using the Benjamini–Hochberg false discovery rate procedure within the relevant family of contrasts. Prior to model fitting, sum-to-zero contrasts were applied using options (contrasts = c(‘contr.sum’, ‘contr.poly’)) to ensure correct Type III decomposition in the presence of interactions.

GI was analyzed using a linear model (LM) with square-root transformation to stabilize variance, with provenance, salinity, seed morphotype and all interactions as fixed effects. Model assumptions were verified by visual inspection of residual plots. Post hoc comparisons were performed as above.

The dose–response analysis was performed using the drc package [38], as described in Section 4.5.

MGT and T50 were analyzed using non-parametric tests, given the substantial number of structurally missing values in replicates with zero germination. The Kruskal–Wallis test was used to assess the effects of provenance and salinity separately, followed by Dunn’s post hoc test with Benjamini–Hochberg correction (dunn.test package) [41] where significant. The effect of seed morphotype on MGT and T50 was tested using the Wilcoxon rank-sum test.

## 5. Conclusions

This study provides, to our knowledge, the first integrated assessment of germination responses across multiple Italian coastal populations of *Salicornia perennans* Willd. subsp. *perennans*, simultaneously evaluating the effects of seed morphotype, population origin and salinity. Seed morphotype emerged as the main determinant of germination performance. Large seeds consistently achieved a higher final germination percentage and germination index than small seeds across populations and salinity treatments, which supports the view that the two morphotypes represent complementary recruitment strategies. Large seeds appear suited to rapid recruitment under favorable conditions, whereas small seeds, characterized by consistently low germination, may contribute to delayed germination and soil seed-bank persistence. Salinity strongly reduced germination percentage and germination index but had no significant effect on MGT or T50 among seeds that successfully germinated. This suggests that salinity acts primarily as a quantitative filter on germination success rather than as a progressive constraint on germination rate. Population origin also influenced germination responses. Although the EC50 values of large seeds were remarkably similar among populations, substantial differences were observed in the maximum germination capacity. In particular, seeds from Lame di San Rossore maintained the highest germination performance across the salinity gradient, reaching 43% final germination at 20 g L^−1^ NaCl, compared with 5% and 1% in Galanchio and Patanella, respectively. These results indicate that population differences are better explained by variation in baseline germination capacity than by variation in salinity sensitivity per se and do not support the expectation that populations from habitats expected to experience stronger or more persistent salinity stress necessarily exhibit greater germination performance under salt stress. Overall, our findings show that germination in *S. perennans* is shaped by the interaction between seed morphotype, population origin and salinity. Maternal environmental effects, differences in seed provisioning and population-specific genetic variation remain plausible explanations for the observed among-population differences, but their relative contribution cannot be resolved within the present study. Future common-garden experiments across multiple generations will be necessary to disentangle these mechanisms.

## Figures and Tables

**Table 1:** Summary of statistical analyses of germination parameters of *Salicornia perennans* Willd. subsp. *perennans*. Final germination percentage (FGP) was analyzed using a generalized linear model (GLM) with quasibinomial error distribution to account for overdispersion, with significance assessed by Type III F-tests. Germination index (GI) was analyzed using a linear model (LM) on square-root-transformed data, with significance assessed by Type III F-tests. Mean germination time (MGT) and time to 50% germination (T50) were analyzed using Kruskal–Wallis tests (H) for provenance and salinity and Wilcoxon rank-sum tests (W) for seed morphotype. Values in bold indicate significant effects (*p* < 0.05); values in italics indicate marginally significant effects (0.05 ≤ *p* < 0.10).

Response Variable/Model	Effect	Statistic	Value	df	df Residual	*p*-Value
**FGP (%) GLM quasibinomial**	Provenance	F	42.84	2	72	**<0.001**
	Salinity	F	74.54	3	72	**<0.001**
	Seed morphotype	F	51.11	1	72	**<0.001**
	Provenance × Salinity	F	2.36	6	72	**0.039**
	Provenance × Seed morphotype	F	7.20	2	72	**0.001**
	Salinity × Seed morphotype	F	1.66	3	72	0.182
	Provenance × Salinity × Seed morphotype	F	1.89	6	72	*0.095*
**GI LM sqrt-transformed**	Provenance	F	32.68	2	72	**<0.001**
	Salinity	F	95.51	3	72	**<0.001**
	Seed morphotype	F	401.61	1	72	**<0.001**
	Provenance × Salinity	F	4.82	6	72	**<0.001**
	Provenance × Seed morphotype	F	26.32	2	72	**<0.001**
	Salinity × Seed morphotype	F	14.73	3	72	**<0.001**
	Provenance × Salinity × Seed morphotype	F	1.23	6	72	0.301
**MGT (days) Kruskal–Wallis**	Provenance	H	6.01	2	-	*0.050*
	Salinity	H	3.93	3	-	0.269
	Seed morphotype	W	1092.5		-	**<0.001**
**T50 (days) Kruskal–Wallis**	Provenance	H	3.81	2	-	0.149
	Salinity	H	5.15	3	-	0.161
	Seed morphotype	W	1024.0		-	**<0.001**

**Table 2:** Parameters of the four-parameter log-logistic (LL.4) dose–response model fitted to final germination percentage (FGP) of *Salicornia perennans* Willd. subsp. *perennans* seeds as a function of NaCl concentration (g L^−1^) for each provenance and seed morphotype (Part A), and pairwise comparisons of EC50 among provenances for large seeds based on the grouped LL.4 model (Part B).

**Part A**							
**Provenance**	**Seed** **morphotype**	**b**	**c**	**d**	**EC50 (g L** **^−^** **^1^** **)**	**95% CI**	**Used** **for inference**
		**slope**	**lower** **asymptote**	**upper** **asymptote**	**inflection point**	**[lower; upper]**	
***Galanchio***	Large	3.35	−0.006	0.873	9.71	[7.78; 12.92]	Yes
	Small	1.28	−0.012	0.261	1.55	[1.49; 1.61]	Descriptive only
***Patanella***	Large	3.56	−0.001	0.449	7.58	[5.40; 9.78]	Yes
	Small	1.51	−0.008	0.263	3.28	[0.09; 6.48]	Descriptive only
***San Rossore***	Large	6.41	0.424	0.960	9.63	[8.24; 10.98]	Yes
	Small	0.09	0.000	0.219	n.e.	n.e.	Descriptive only
**Part B**							
**Comparison (EC50 ratio)**				**Estimate**	**SE**	**t-value**	***p*** **-value**
Galanchio/Patanella				1.281	0.224	1.254	0.210
Galanchio/San Rossore				1.009	0.114	0.077	0.939
Patanella/San Rossore				0.788	0.133	−1.602	0.109

## Data Availability

The data supporting the findings of this study are available within the article and its Supplementary Materials. Additional information may be obtained from the corresponding author upon reasonable request.

## Supplementary Materials

The following supporting information can be downloaded at: https://www.mdpi.com/article/10.3390/plants15152285/s1, Table S1: Mean seed length and width (±SE) of large and small seeds of *S. perennans* subsp. *perennans* from Galanchio, Patanella and San Rossore. Table S2: Germination parameters (means ± SE; Part A), post hoc pairwise comparisons for FGP (Parts B–C) and GI (Parts D–E), and complete mean-separation results across all provenance × salinity combinations for both FGP and GI, analyzed separately per seed morphotype (Part F). Table S3: Monthly climate data for the three study sites (Bocca d’Arno, Coltano and Orbetello stations; SIR Toscana, 2013–2024). Table S4: Robustness checks for dose–response models fitted to FGP of large seeds, comparing LL.4, LL.3 and W1.4 model families. Figure S1: EC50 estimates with 95% confidence intervals for large- and small-seed morphotypes of *S. perennans* subsp. *perennans* from three Tuscan coastal populations.
