# Supplementary material for: Seed Morphotype and Population Origin Shape Germination Responses of Three Salicornia perennans Willd. subsp. perennans Populations Under Different NaCl Concentrations
Source: Plants (Basel). 2026 Jul 26;15(15):2285. doi: 10.3390/plants15152285 (PMC13468056; doi:10.3390/plants15152285)
Supplement: Supplementary file 1 [file plants-15-02285-s001.zip › Table S1_seeds.pdf]

Table S1. Mean seed length and width (± SE) of large (L) and small (s) seeds of *Salicornia perennans* subsp. *perennans* collected from the Galanchio, Patanella and San Rossore populations. Length and width were measured from digital images using ImageJ software and are expressed in millimetres (mm). Large seeds were consistently longer and wider than small seeds in all populations, with the Patanella population producing the largest seeds for both morphotypes.

Population	Seed type	Length (mm)	Width (mm)
Galanchio	L	1.271 ± 0.021	0.5246 ± 0.004
	s	0.904 ± 0.003	0.4156 ± 0.005
Patanella	L	1.749 ± 0.011	0.9627 ± 0.002
	s	0.967 ± 0.004	0.5547 ± 0.005
San Rossore	L	1.312 ± 0.002	0.7762 ± 0.002
	s	0.944 ± 0.001	0.4967 ± 0.003
