# Supplementary material for: Seed Morphotype and Population Origin Shape Germination Responses of Three Salicornia perennans Willd. subsp. perennans Populations Under Different NaCl Concentrations
Source: Plants (Basel). 2026 Jul 26;15(15):2285. doi: 10.3390/plants15152285 (PMC13468056; doi:10.3390/plants15152285)
Supplement: Supplementary file 1 [file plants-15-02285-s001.zip › Table S2 a, b,c,d,e,f.pdf]

Table S2. Supplementary germination data for *Salicornia perennans* from three Tuscan coastal populations. Values are reported as mean ± SE.

Part A. Germination parameters (mean ± SE) across four salinity treatments and two seed morphotypes.

Provenance	Salinity (g/L)	Salinity (mM) NaCl	Seed morphotype	FGP (%)	GI	MGT (days)	n MGT	T50 (days)	n T50
Galanchio	0	0	Small	26 ± 2.58	1.11 ± 0.13	6.10 ± 0.30	4	5.68 ± 0.21	4
			Large	89 ± 4.43	5.49 ± 0.37	4.19 ± 0.12	4	3.55 ± 0.05	4
	5	85.6	Small	4 ± 2.83	0.16 ± 0.11	6.33 ± 0.33	2	5.62 ± 0.12	2
			Large	75 ± 5.51	3.91 ± 0.28	4.97 ± 0.27	4	4.46 ± 0.34	4
	10	171.1	Small	2 ± 1.15	0.06 ± 0.04	8.00 ± 0.00	2	7.50 ± 0.00	2
			Large	45 ± 6.61	1.87 ± 0.33	6.48 ± 0.28	4	6.02 ± 0.44	4
	20	342.2	Small	0 ± 0.00	0.00 ± 0.00	n.c.	0	n.c.	0
			Large	5 ± 1.91	0.23 ± 0.08	5.67 ± 1.33	3	5.17 ± 1.33	3
Patanella	0	0	Small	26 ± 8.08	1.01 ± 0.34	6.62 ± 0.36	4	6.15 ± 0.40	4
			Large	45 ± 5.00	2.44 ± 0.11	4.94 ± 0.57	4	4.40 ± 0.69	4
	5	85.6	Small	9 ± 2.52	0.37 ± 0.08	5.88 ± 0.52	4	5.25 ± 0.43	4
			Large	34 ± 5.03	1.77 ± 0.25	5.27 ± 0.30	4	4.67 ± 0.47	4
	10	171.1	Small	4 ± 2.83	0.20 ± 0.14	5.00 ± 0.00	2	4.50 ± 0.00	2
			Large	13 ± 1.00	0.75 ± 0.10	5.23 ± 0.79	4	5.58 ± 1.12	4
	20	342.2	Small	1 ± 1.00	0.06 ± 0.06	4.00	1	3.50	1
			Large	1 ± 1.00	0.05 ± 0.05	5.00	1	4.50	1
San Rossore	0	0	Small	14 ± 2.58	0.57 ± 0.12	6.53 ± 0.35	4	6.08 ± 0.28	4
			Large	96 ± 1.63	4.68 ± 0.18	5.37 ± 0.19	4	5.05 ± 0.26	4
	5	85.6	Small	11 ± 3.00	0.43 ± 0.11	6.62 ± 0.24	4	5.88 ± 0.31	4
			Large	95 ± 1.91	4.64 ± 0.12	5.35 ± 0.08	4	5.07 ± 0.14	4
	10	171.1	Small	13 ± 4.43	0.49 ± 0.17	6.83 ± 0.17	3	6.17 ± 0.17	3
			Large	66 ± 4.76	2.84 ± 0.21	5.91 ± 0.07	4	5.48 ± 0.03	4
	20	342.2	Small	6 ± 3.83	0.22 ± 0.14	7.00 ± 0.00	2	6.00 ± 0.00	2

			Large	43 ± 8.54	1.75 ± 0.35	6.24 ± 0.08	4	5.67 ± 0.06	4

FGP: final germination percentage; GI: germination index; MGT: mean germination time; T50: time to 50% germination. FGP and GI values are means ± SE of four replicates. MGT and T50 were calculated only for replicates with at least one germinated seed; zero-germination replicates were excluded from both MGT and T50 calculations. The corresponding number of replicates is reported as n MGT and n T50. n.c.: not calculable. Single-replicate values are reported without SE.

Part B. Post-hoc pairwise comparisons of FGP between seed morphotypes (small vs large) for each provenance and salinity level (GLM quasibinomial, emmeans, BH correction). Green: $p < 0.05$; yellow: $0.05 \leq p < 0.10$.

Provenance	Salinity (Nacl g/L)	Odds ratio (small/large)	SE	z	p-value (BH)
Galanchio	0	0.043	0.019	-7.026	<0.001
	5	0.014	0.009	-6.714	<0.001
	10	0.025	0.021	-4.374	<0.001
	20	<0.001	0.000	-0.011	0.991
Patanella	0	0.429	0.148	-2.445	0.015
	5	0.192	0.089	-3.555	<0.001
	10	0.279	0.187	-1.901	0.057
	20	1.000	1.620	0.000	1.000
San Rossore	0	0.007	0.005	-7.492	<0.001
	5	0.007	0.004	-7.918	<0.001
	10	0.077	0.032	-6.183	<0.001
	20	0.085	0.045	-4.650	<0.001

Odds ratio (small/large): a ratio < 1 indicates higher FGP in large seeds. Contrasts involving near-zero germination in both morphotypes, particularly Galanchio and Patanella at 20 g/L NaCl, are biologically uninformative and should not be interpreted as evidence of morphotype differences.

Part C. Post-hoc pairwise comparisons of FGP among provenances for each salinity level and seed morphotype (GLM quasibinomial, emmeans, BH correction). Expressed as odds ratios. Green: $p < 0.05$.

Salinity (g/L)	Seed morphotype	Contrast	Odds ratio	SE	z	p-value (BH)
0	Small	Galanchio / Patanella	1.000	0.367	0.000	1.000
		Galanchio / San Rossore	2.158	0.902	1.841	0.099
		Patanella / San Rossore	2.158	0.902	1.841	0.099
5		Galanchio / Patanella	0.421	0.296	-1.229	0.329
		Galanchio / San Rossore	0.337	0.231	-1.588	0.329
		Patanella / San Rossore	0.800	0.431	-0.414	0.679
10		Galanchio / Patanella	0.490	0.489	-0.715	0.475
		Galanchio / San Rossore	0.137	0.120	-2.263	0.071
		Patanella / San Rossore	0.279	0.187	-1.901	0.086
20		Galanchio / Patanella	<0.001	0.000	-0.010	0.992
		Galanchio / San Rossore	<0.001	0.000	-0.012	0.992
		Patanella / San Rossore	0.158	0.196	-1.488	0.411
0	Large	Galanchio / Patanella	9.889	4.250	5.337	<0.001
		Galanchio / San Rossore	0.337	0.231	-1.588	0.112
		Patanella / San Rossore	0.034	0.021	-5.417	<0.001
5		Galanchio / Patanella	5.824	2.070	4.952	<0.001

		Galanchio / San Rossore	0.158	0.092	-3.160	0.002
		Patanella / San Rossore	0.027	0.016	-6.281	<0.001
10		Galanchio / Patanella	5.476	2.230	4.166	<0.001
		Galanchio / San Rossore	0.422	0.140	-2.606	0.009
		Patanella / San Rossore	0.077	0.032	-6.183	<0.001
20		Galanchio / Patanella	5.211	6.550	1.314	0.189
		Galanchio / San Rossore	0.070	0.040	-4.670	<0.001
		Patanella / San Rossore	0.013	0.016	-3.700	<0.001

A ratio > 1 indicates higher FGP in the numerator provenance. BH correction was applied across three pairwise comparisons within each salinity × seed morphotype combination.

Part D. Post-hoc pairwise comparisons of GI between seed morphotypes (small vs large) for each provenance and salinity level (LM on sqrt-transformed GI, emmeans, BH correction). Green: $p < 0.05$.

Provenance	Salinity (g/L)	Estimate (small - large)	SE	t	p-value (BH)
Galanchio	0	-1.290	0.165	-7.818	<0.001
	5	-1.702	0.165	-10.316	<0.001
	10	-1.174	0.165	-7.115	<0.001
	20	-0.412	0.165	-2.495	0.015
Patanella	0	-0.615	0.165	-3.726	<0.001
	5	-0.722	0.165	-4.377	<0.001
	10	-0.555	0.165	-3.362	0.001
	20	0.013	0.165	0.080	0.937
San Rossore	0	-1.422	0.165	-8.617	<0.001
	5	-1.523	0.165	-9.232	<0.001
	10	-1.077	0.165	-6.530	<0.001
	20	-0.976	0.165	-5.915	<0.001

Estimates represent differences between seed morphotypes, calculated as small minus large, on the square-root-transformed GI scale. Negative values indicate higher GI in large seeds. SE = 0.165 for all contrasts due to the balanced design. The contrast for Patanella at 20 g/L should be interpreted with caution because GI was near zero in both morphotypes.

Part E. Post-hoc pairwise comparisons of GI among provenances for each salinity level and seed morphotype (LM on sqrt-transformed GI, emmeans, BH correction). Estimates on sqrt scale. Green: $p < 0.05$; yellow: $0.05 <= p < 0.10$.

Salinity (g/L)	Seed morphotype	Contrast	Estimate	SE	t	p-value (BH)
0	Small	Galanchio - Patanella	0.102	0.165	0.619	0.538
		Galanchio - San Rossore	0.307	0.165	1.861	0.200
		Patanella - San Rossore	0.205	0.165	1.243	0.327
5		Galanchio - Patanella	-0.329	0.165	-1.992	0.075
		Galanchio - San Rossore	-0.360	0.165	-2.180	0.075
		Patanella - San Rossore	-0.031	0.165	-0.188	0.851
10		Galanchio - Patanella	-0.129	0.165	-0.780	0.438
		Galanchio - San Rossore	-0.426	0.165	-2.584	0.035

		Patanella - San Rossore	-0.298	0.165	-1.804	0.113
20		Galanchio - Patanella	-0.125	0.165	-0.758	0.451
		Galanchio - San Rossore	-0.326	0.165	-1.976	0.156
		Patanella - San Rossore	-0.201	0.165	-1.218	0.341
0	Large	Galanchio - Patanella	0.777	0.165	4.711	**<0.001**
		Galanchio - San Rossore	0.175	0.165	1.061	0.292
		Patanella - San Rossore	-0.602	0.165	-3.649	**0.001**
5		Galanchio - Patanella	0.651	0.165	3.948	**<0.001**
		Galanchio - San Rossore	-0.181	0.165	-1.095	0.277
		Patanella - San Rossore	-0.832	0.165	-5.043	**<0.001**
10		Galanchio - Patanella	0.491	0.165	2.974	**0.006**
		Galanchio - San Rossore	-0.330	0.165	-1.998	**0.050**
		Patanella - San Rossore	-0.820	0.165	-4.972	**<0.001**
20		Galanchio - Patanella	0.300	0.165	1.817	0.073
		Galanchio - San Rossore	-0.890	0.165	-5.396	**<0.001**
		Patanella - San Rossore	-1.190	0.165	-7.213	**<0.001**

Estimates represent differences between provenances on the square-root-transformed GI scale. A positive value indicates higher GI in the first-named provenance. SE = 0.165 for all contrasts due to the balanced design. BH correction was applied across three pairwise comparisons within each salinity × seed morphotype combination.

Part F. Mean separation for the provenance × salinity interaction for final germination percentage (FGP) and germination index (GI), performed separately for each seed morphotype.

Provenance	NaCl	FGP Large	FGP Small	GI Large	GI Small
Galanchio	0	89 ± 4.43 a	26 ± 2.58 a	5.49 ± 0.37 a	1.11 ± 0.13 a
Galanchio	5	75 ± 5.51 b	4 ± 2.83 b	3.91 ± 0.28 bc	0.16 ± 0.11 cd
Galanchio	10	45 ± 6.61 c	2 ± 1.15 b	1.87 ± 0.33 de	0.06 ± 0.04 d
Galanchio	20	5 ± 1.91 de	0 ± 0.00 ab†	0.23 ± 0.08 g	0.00 ± 0.00 d
Patanella	0	45 ± 5.00 c	26 ± 8.08 a	2.44 ± 0.11 de	1.01 ± 0.34 ab
Patanella	5	34 ± 5.03 c	9 ± 2.52 b	1.77 ± 0.25 e	0.37 ± 0.08 bc
Patanella	10	13 ± 1.00 d	4 ± 2.83 b	0.75 ± 0.10 f	0.20 ± 0.14 cd
Patanella	20	1 ± 1.00 e	1 ± 1.00 b	0.05 ± 0.05 g	0.06 ± 0.06 d
San Rossore	0	96 ± 1.63 a	14 ± 2.58 ab	4.68 ± 0.18 ab	0.57 ± 0.12 ab
San Rossore	5	95 ± 1.91 a	11 ± 3.00 ab	4.64 ± 0.12 ab	0.43 ± 0.11 bc
San Rossore	10	66 ± 4.76 b	13 ± 4.43 ab	2.84 ± 0.21 cd	0.49 ± 0.17 bc
San Rossore	20	43 ± 8.54 c	6 ± 3.83 b	1.75 ± 0.35 e	0.22 ± 0.14 cd
