# Supplementary material for: Seed Morphotype and Population Origin Shape Germination Responses of Three Salicornia perennans Willd. subsp. perennans Populations Under Different NaCl Concentrations
Source: Plants (Basel). 2026 Jul 26;15(15):2285. doi: 10.3390/plants15152285 (PMC13468056; doi:10.3390/plants15152285)
Supplement: Supplementary file 1 [file plants-15-02285-s001.zip › TableS3_Climate.pdf]

Table S3. Monthly and annual climate data for the three study sites, derived from daily records of the nearest SIR Toscana meteorological stations over the period 2013–2024.

Bocca d'Arno station (Lat 43.681°N, Lon 10.280°E, 1 m a.s.l.) for Lame di San Rossore; Coltano station (Lat 43.638°N, Lon 10.391°E, 5 m a.s.l.) for Galanchio; Orbetello station (Lat 42.434°N, Lon 11.203°E, 0 m a.s.l.) for Patanella. Orange shading: highest monthly value; blue shading: lowest monthly value. Annual column shows mean annual precipitation (mm) or mean annual temperature (°C).

Precipitation (mm)	Jan	Feb	Mar	Apr	May	Jun	Jul	Aug	Sep	Oct	Nov	Dec	Annual mean
Lame di San Rossore (Bocca d'Arno station)	96	96	72	52	57	**31**	42	41	81	118	**133**	91	910
Galanchio (Coltano station)	98	81	71	51	58	37	**34**	44	75	111	**126**	92	879
Patanella (Orbetello station)	44	51	40	24	27	20	**7**	**7**	46	71	**93**	68	508

Mean temperature (°C)	Jan	Feb	Mar	Apr	May	Jun	Jul	Aug	Sep	Oct	Nov	Dec	Annual mean
Lame di San Rossore (Bocca d'Arno station)	**8.5**	9.4	10.9	13.6	17.1	21.5	**24.1**	**24.1**	21.0	17.6	13.2	9.7	15.9
Galanchio (Coltano station)	**7.9**	9.1	11.0	13.8	17.3	21.8	24.6	**24.8**	21.2	17.4	12.6	8.8	15.9
Patanella (Orbetello station)	**9.4**	10.5	12.0	14.6	18.1	22.6	**25.2**	**25.2**	21.9	18.6	14.4	10.7	17.0

Mean maximum temperature (°C)	Jan	Feb	Mar	Apr	May	Jun	Jul	Aug	Sep	Oct	Nov	Dec	Annual mean
Lame di San Rossore (Bocca d'Arno station)	**12.2**	13.1	14.9	17.7	20.7	25.4	28.0	**28.2**	25.1	21.5	16.8	13.4	19.8
Galanchio (Coltano station)	**12.1**	13.8	16.3	19.6	22.6	27.6	30.6	**31.1**	26.8	22.2	16.6	12.9	21.1
Patanella (Orbetello station)	**12.3**	13.2	14.6	17.3	20.4	25.0	27.8	**28.0**	24.7	21.2	17.0	13.7	19.6

Mean minimum temperature (°C)	Jan	Feb	Mar	Apr	May	Jun	Jul	Aug	Sep	Oct	Nov	Dec	Annual mean
Lame di San Rossore (Bocca d'Arno station)	**4.8**	5.6	6.8	9.5	13.5	17.6	**20.2**	20.0	16.9	13.7	9.5	5.9	12.1

Galanchio (Coltano station)	**3.7**	4.5	5.6	7.9	12.0	15.9	**18.5**	**18.5**	15.6	12.6	8.5	4.8	10.7
Patanella (Orbetello station)	**6.5**	7.8	9.4	12.0	15.7	20.1	**22.5**	**22.5**	19.1	16.0	11.7	7.8	14.3

Source: Servizio Idrologico Regionale della Toscana (SIR Toscana), www.sir.toscana.it. Data retrieved 2025.
