# Supplementary material for: Seed Morphotype and Population Origin Shape Germination Responses of Three Salicornia perennans Willd. subsp. perennans Populations Under Different NaCl Concentrations
Source: Plants (Basel). 2026 Jul 26;15(15):2285. doi: 10.3390/plants15152285 (PMC13468056; doi:10.3390/plants15152285)
Supplement: Supplementary file 1 [file plants-15-02285-s001.zip › TableS4_DoseResponse_Robustness_BW.pdf]

Table S4. Robustness checks for the dose-response models fitted to final germination percentage (FGP) of large seeds, conducted in response to reviewer comments on the reliability of the EC50 and upper-asymptote (d) estimates.

Part A. Comparison between the four-parameter (LL.4, lower asymptote free) and three-parameter (LL.3, lower asymptote fixed at zero) log-logistic models.

Provenance	AIC LL.4	AIC LL.3	ΔAIC (LL.3−LL.4)	EC50 (g L^{-1}) LL.4	EC50 (g L^{-1}) LL.3	d (%) LL.4 / LL.3
Galanchio	124.05	123.62	-0.44	12.20	9.89	88.96 / 87.00
Patanella	114.25	112.26	-1.99	7.34	7.33	44.89 / 44.90
San Rossore	124.80	126.16	1.36	9.61	16.64	96.02 / 98.54

ΔAIC = AIC(LL.3) − AIC(LL.4); positive values favour LL.4. Differences below 2 units indicate that the two model formulations describe the data comparably well. d: upper asymptote, representing maximum germination capacity (%).

Part B. Comparison between the log-logistic (LL.4) and Weibull type I (W1.4) dose-response models, representing two distinct functional forms.

Provenance	AIC LL.4	AIC W1.4	EC50 (g L^{-1}) LL.4	EC50 (g L^{-1}) W1.4	d (%) LL.4	d (%) W1.4
Galanchio	124.05	124.05	12.20	12.27	88.96	89.01
Patanella	114.25	114.13	7.34	8.89	44.89	45.00
San Rossore	124.80	124.80	9.61	10.34	96.02	96.00

Parameter estimates were highly consistent between model families for both EC50 and d in all three populations, indicating that the results are not dependent on the choice of dose-response function.

Part C. Ranking of the three populations by EC50 and by maximum germination capacity (d), compared between the log-logistic (LL.4) and Weibull (W1.4) model families.

Comparison	Ranking (both model families)
**EC50 (lowest → highest)**	Patanella < San Rossore < Galanchio
**d, maximum germination capacity (highest → lowest)**	San Rossore > Galanchio > Patanella

The ranking of populations was identical between the two model families for both parameters, confirming that the pattern of similar salinity thresholds (EC50) but substantially different maximum germination capacities (d) is robust to the choice of dose-response model.
